# Leptin Promotes Wound Healing in the Skin

**DOI:** 10.1371/journal.pone.0121242

**Published:** 2015-03-23

**Authors:** Susumu Tadokoro, Shinji Ide, Reiko Tokuyama, Hirochika Umeki, Seiko Tatehara, Shiki Kataoka, Kazuhito Satomura

**Affiliations:** Department of Oral Medicine and Stomatology, Tsurumi University School of Dental Medicine, 2-1-3 Tsurumi, Tsurumi-ku, Yokohama, Kanagawa 230-8501, Japan; University of Tennessee, UNITED STATES

## Abstract

**Introduction:**

Leptin, a 16 kDa anti-obesity hormone, exhibits various physiological properties. Interestingly, skin wound healing was proven to delay in leptin-deficient *ob/ob* mice. However, little is known on the mechanisms of this phenomenon. In this study, we attempted to elucidate a role of leptin in wound healing of skin.

**Methods:**

Immunohistochemical analysis was performed to confirm the expression of the leptin receptor (Ob-R) in human and mouse skin. Leptin was topically administered to chemical wounds created in mouse back skin along with sustained-release absorbable hydrogel. The process of wound repair was histologically observed and the area of ulceration was measured over time. The effect of leptin on the proliferation, differentiation and migration of human epidermal keratinocytes was investigated.

**Results:**

Ob-R was expressed in epidermal cells of human and mouse skin. Topical administration of leptin significantly promoted wound healing. Histological analysis showed more blood vessels in the dermal connective tissues in the leptin-treated group. The proliferation, differentiation/function and migration of human epidermal keratinocytes were enhanced by exogenous leptin.

**Conclusion:**

Topically administered leptin was proven to promote wound healing in the skin by accelerating proliferation, differentiation/function and migration of epidermal keratinocytes and enhancing angiogenesis around the wounded area. These results strongly suggest that topical administration of leptin may be useful as a treatment to promote wound healing in the skin.

## Introduction

Leptin, the product of *ob(obese)* gene, is a 16 kDa non-glycosylated polypeptide anti-obesity hormone mainly produced and secreted by adipose tissues [1]. It influences body weight homeostasis through its effects on food intake and energy expenditure by negative feedback at the hypothalamic nuclei [2]. In addition to these effects through the central nervous system, recent studies have demonstrated that leptin has various physiological roles in lipid metabolism [3], hematopoiesis [4], thermogenesis [5], ovarian function [6], bone formation [7,8] and angiogenesis [9,10]. Another series of studies have also demonstrated that this hormone is produced by some tissues such as placenta [11], stomach [12], skeletal muscles [13], tooth germ [14], brain and pituitary gland [15,16] other than adipose tissue. The leptin receptor (Ob-R) is also expressed in various tissues including the hypothalamus [17,18], adipose tissue [19], skeletal muscle [20], hepatocytes [19,21], and epithelial cells [22,23]. The multifuncitionality of leptin is considered to come from this wide distribution of production sites and target cells.

Some past studies unveiled the effect of leptin on would healing by demonstrating that leptin acted as an autocrine/paracrine regulator in the wounded sites [24]. Another study also showed that skin would healing delayed in leptin deficient *ob/ob* mice and that exogenously administered leptin restored this delayed wound healing by enhancing re-epithelialization of the wound in these mice [22]. Moreover, in the pervious study, we demonstrated that local administration of leptin could also promote wound healing in the oral mucosa [23]. These findings strongly suggest the possibility that leptin could be a potential medicine for promoting wound healing in both skin and mucosa. However, all such literatures refer to whole body dosage administered intraperitoneally, and even when administered locally, the leptin must have been administered every day. Judging from the fact that leptin is a multifunctional and potent systemic hormone, a systemic administration or multiple local administration of leptin may cause some sort of adverse effect in its clinical application. In addition, local and single administration of medicine could be more advantageous for the lowering of patients’ distress in some cases in clinical practice. From these points of view, it is considered significant to examine whether local and single-dose administration of leptin can also exert its influence on the wound healing. Hence, in this study, we investigated whether leptin exerted a promotive influence on the skin would healing even when administered with a low single dosage and one time by using MedGel, a bioabsorbable hydrogel used for a drug delivery system (DDS).

## Materials and Methods

### Human tissue samples

The study was approved by the Research Ethics Review Committee of Tsurumi University School of Dental Medicine (approval number: 1048). After written informed consent was obtained, small pieces of skin were obtained from excess portion of free skin grafts of the patients who underwent reconstructive surgery. The tissues were fixed with 10 N Mildform (Wako Pure Chemical Industries Ltd, Osaka, Japan), and embedded in paraffin. Sections were cut at 5 μm thickness, deparaffinized and stained with hematoxylin and eosin.

### Creation of chemical wounds

**Fig 1:**
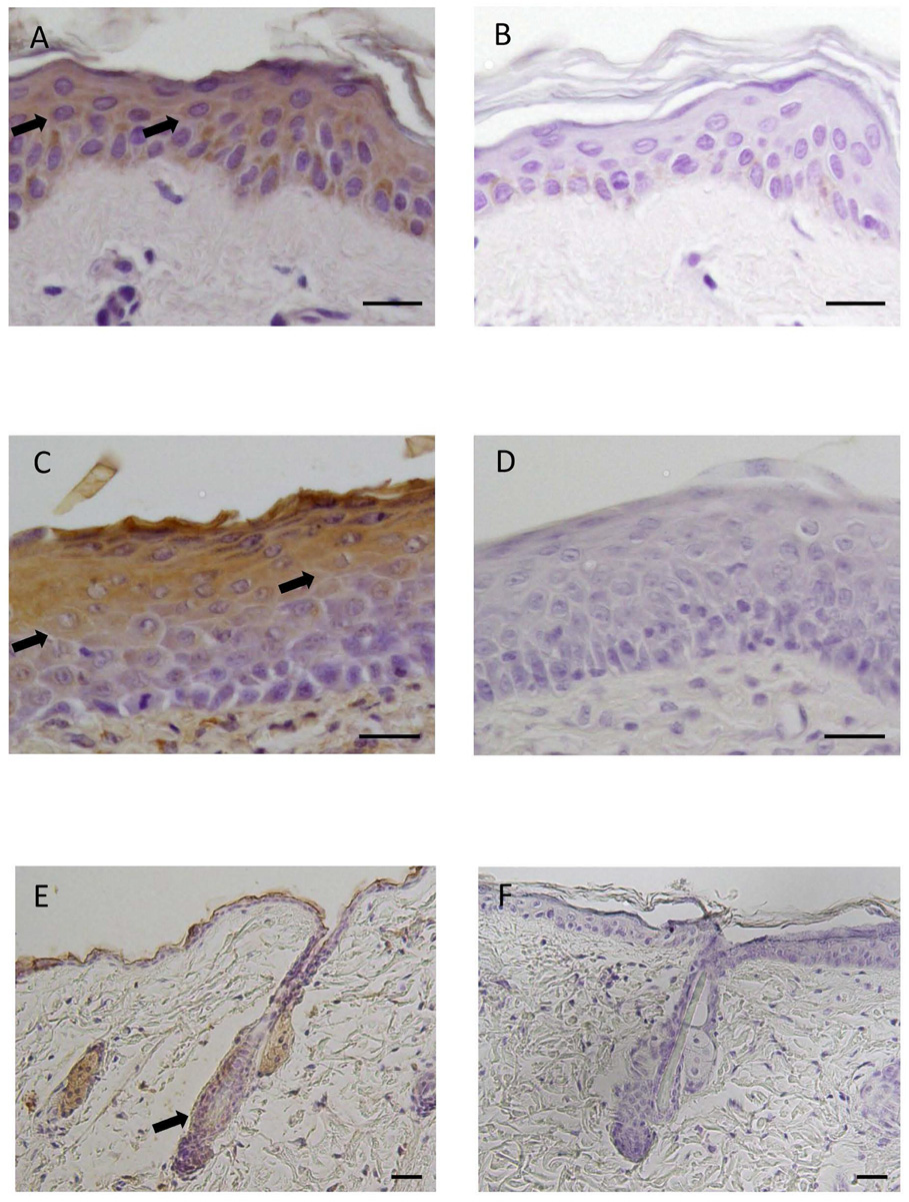
Immunohistochemical localization of Ob-R in human and mouse skin. (A) Immunohistochemical staining for Ob-R. Ob-R was expressed in epidermal cells of humen skin (arrows). DAB and hematoxylin staining. (B) Negative control. (C) Immunohistochemical staining for Ob-R. Epidermal cells of mouse skin, particularly prickle/gananular cells, were positive for Ob-R (arrows). DAB and hematoxylin staining. (D) Negative control. (E) Immunohistochemical staining for Ob-R in mouse skin. Ob-R was also expressed in hair follicles (arrow). DAB and hematoxylin staining. (F) Negative control. Scale bars = 20 μm.

The animal care and experimental protocols were approved by the Committee on the Ethics of Animal Experiments of Tsurumi University (approval number: 24A064). All surgery was performed under general anesthesia by intraperitoneal injection of 40 mg/kg pentobarbital sodium, and all efforts were made to minimize suffering. Eighteen 6-week-old male ICR mice were obtained from CLEA Japan, Inc. (Tokyo, Japan), fed a normal diet and maintained under a 12-hour-light/12-hour-dark cycle at 22°C. Chemical wounds were created on the back skin by applying two pieces of filter paper (12x12 mm each) soaked with 20% sodium hypochlorite for 5 minutes (Fig. 1). Wound formation was verified next day, and the wounds were covered with 15 g (12 x 12 x 1 mm) of MedGel (MedGEL Corp., Tokyo, Japan) containing 10 μL of 100 ng/mL leptin (R&D Systems, Minneapolis, USA) or phosphate-buffered saline (PBS). The MedGel was fixed in position by using a wound-protection dressing (ConvaTec Inc., Greensboro, NC, USA). The ulcer size was measured on day 4 and day 8 after wound formation, and the skin tissues around the wound were obtained for histological analysis. Excised tissue was fixed with 10 N Mildform (Wako) and embedded in paraffin. Sections were cut at 8 μm thickness, deparaffinized and stained with hematoxylin and eosin.

### Immunohistochemical analysis

Tissue sections of human and mouse skin were transferred onto poly-l-lysine-coated glass slides (Matsunami Glass, Osaka, Japan). After deparaffinization with xylene and rehydration with descending concentrations of ethanol, endogenous peroxidase was blocked by treatment with 3% H_2_O_2_ in methanol for 1 h at room temperature (RT). After treatment with 10% normal rabbit serum at RT for 10 min, sections were incubated with goat anti-Ob-R antibody (Santa Cruz Biotechnology, Inc., CA, USA) diluted 1:750 in PBS (pH 7.4) containing 1% bovine serum albumin at 4°C overnight. After washing with PBS, the localization of Ob-R was visualized using a Histofine SAB-PO (G) kit (Nichirei Corporation, Tokyo, Japan) and a 3,3′-diaminobenzidine (DAB) substrate kit (Nichirei Corporation). Sections were counterstained with hematoxylin and mounted. The specificity of the immunoreaction was confirmed by incubation with normal goat IgG or normal goat serum instead of the primary antibody.

Some sections of mouse skin around the wound were deparaffinized, and endogenous peroxidase was blocked in the manner described above. After treatment with 10% normal goat serum at RT for 10 min, the sections were incubated with rabbit anti-CD31 antibody (Abcam, Cambridge, UK) diluted 1:100 in PBS (pH 7.4) containing 1% bovine serum albumin at 4°C overnight. After rinsing with PBS, the localization of CD31, a marker molecule of endothelial cell, was visualized using a Histofine Max-PO (R) kit (Nichirei Corporation) and a 3,3′-diaminobenzidine (DAB) substrate kit (Nichirei Corporation). Sections were counterstained with hematoxylin and mounted. The specificity of the immunoreaction was confirmed by incubation with normal rabbit IgG or normal rabbit serum instead of the primary antibody.

### Histometric analysis

A histometric analysis was performed using tissue sections at day 4 and day 8 after wound creation. Five mice were sacrificed and 5 tissue sections were made for each sample. After immunohistochemical staining, 5 fields (850 μm × 640 μm each) in the dermal connective tissue beneath the ulcer were arbitrarily selected and the CD31-positive cells were counted.

### Cell culture

Normal human epidermal keratinocytes (Lonza Ltd, Basel, Switzerland) were cultured in KGM-Gold Basal Medium containing 0.4% bovine pituitary extract (BPE), 0.1% recombinant human epidermal growth factor (hEGF), 0.1% bovine insulin, 0.1% hydrocortisone, transferring, 0.05% epinephrine, 0.1% gentamicin, and 0.1% amphotericin-B (Lonza). The culture was maintained at 37°C in humidified atmosphere of 5% CO_2_ in air and the medium was changed twice a week.

### Cell proliferation assay

The effect of leptin on the proliferation of human epidermal keratinocytes was analyzed using a crystal violet staining method [25]. In brief, cells were plated at a cell density of 4 x 10^3^ cells per well in 12-well culture plates. Cells were treated with various concentrations (0, 10, 50, 100 and 200 ng/mL) of leptin (R&D Systems) for 1, 7 and 14 days. On each scheduled day, cells were rinsed with PBS and fixed with 1% glutaraldehyde in PBS overnight at 4°C. The cells were then stained with 0.02% crystal violet in deionized water for 30 min at RT. After several rinses with distilled water, crystal violet bound to cells was extracted by overnight incubation with 500 μL/well of 70% ethanol at 4°C. Absorbance was measured at 570 nm using a microplate reader Model 680 (Bio-Rad, California, USA).

### Semi-quantitative RT-PCR analysis

Human epidermal keratinocytes were seeded into 60 mm petri dishes at a cell density of 4 x 10^4^ cells/dish, and cultured until they reached confluence. The day at confluence was designated as day 0. We first confirmed the expression of Ob-R mRNA in the cells by reverse transcription polymerase chain reaction (RT-PCR) analysis. Thereafter, the cells were treated with or without 100 ng/ml of leptin for various periods. The expression of mRNA encoding *Cytokeratin 13*, *Cytokeratin 14*, *Transglutaminase I* and *G3PDH* was examined by semi-quantitative RT-PCR analysis. In brief, on each scheduled day, total RNA was extracted from the cells using TRIzol reagent (Invitrogen, Carlsbad, USA), and cDNA was generated from 1 μg of the total RNA using SuperScript III First-Strand Synthesis System (Invitrogen). The PCR amplification was carried out in a 50 μL reaction mixture using 1.1x ReddyMix PCR Master Mix (1.5 mM MgCl_2_: ABgene, Thermo Scientific, Waltham, USA). Conditions and primer sequences for PCR amplification are shown in Table 1. The *G3PDH* gene was used as an internal control for the quantity and quality of cDNA. The PCR products were analyzed by ethidium bromide staining after separation by electrophoresis through a 2% agarose gel.

**Table 1:** Oligonucleotide primers used in RT-PCR.

Primers	Sequence	Size (bp)
G3PDH	F: 5’-acc aca gtc cat gcc atc ac-3’	451
	R: 5’-tcc acc acc ctg ttg ctg ta-3’	
Ob-R	F: 5’-gct att ttg gga aga tgt-3’	499
	R: 5’-tgc ctg ggc ctc tat ctc-3’	
Cytokeratin 13	F: 5’-ttc cta cct gga gaa ggt gcg c-3’	310
	R: 5’-aca gtg agc tca tcc agc acc c-3’	
Cytokeratin 14	F: 5’-tgg tgg cct tgg tac tgg ctt g-3’	285
	R: 5’-gca ttg tcc act gtg gct gtg ag-3’	
Transgkutaminase I	F: 5’-atg gat ggg cca cgt tcc gat-3’	479
	R: 5’-tca gag gat tca tag gtc cgg-3'	

*F*: forward, *R*: reverse.

### Real-time RT-PCR analysis

The expression of mRNA encoding *Cytokeratin 13*, *Cytokeratin 14* and *Transglutaminase I* and *G3PDH* in human epidermal keratinocytes was also examined by real-time PCR. PCR was performed with SYBR Premix Ex Taq (Takara Bio Inc., Shiga, Japan) using an Applied Biosystems StepOne Real-Time PCR System (Applied Biosystems Inc., Carlsbad, CA, USA). Conditions and primer sequences for PCR amplification are shown in Table 2. The *G3PDH* gene was used as an internal control for the quantity and quality of cDNA. The expression levels of genes were analyzed based on the ΔΔ ct method. Results were presented as fold changes of gene expression level ± SD.

**Table 2:** Oligonucleotide primers used in real-time RT-PCR.

Primers	Sequence	Size (bp)
G3PDH	F: 5’-gag tca acg gat ttg gtc g-3’	248
	R: 5’-ttg att ttg gag gga tct cg-3’	
Cytokeratin 13	F: 5’-cga gag cct gaa tga aga gc-3’	205
	R: 5’-gtg gaa cca tcc ctc agc at-3’	
Cytokeratin 14	F: 5’-ttc tga ac gaga tgc gtg ac-3’	189
	R: 5’-gca gct caa tct cca ggt tc-3’	
Transglutaminase I	F: 5’-cat caa gaa tgg cct ggt ct-3’	110
	R: 5’-caa tct tga agc tgc cat ca-3’	

*F*: forward, *R*: reverse.

### Western blot analysis

Human epidermal keratinocytes were lysed in RIPA buffer (10 mM Tris-HCl, 1% NP-40, 0.1% SDS, 150 mM NaCl and 1 mM EDTA) containing protease inhibitor cocktail (Santa Cruz Biotechnology). The homogenates were centrifuged at 10,000 rpm, 4°C for 20 min. The supernatants were mixed with NuPAGE LDS sample buffer (Invitrogen) and heated for 3 min at 100°C. The samples were electrophoretically separated by the NuPAGE System (Invitrogen) using a 4–12% Bis-Tris gel, and electroblotted onto a PVDF membrane using iBlot Dry Blotting System (Invitrogen). The membrane was blocked with Western Breeze Blocking Solution (Invitrogen) for 30 min at RT, and incubated with goat anti-Ob-R antibody (1:250; R&D Systems) or rabbit anti-β-actin antibody (1:1000; Biolegend, California, USA) for 1 h at RT. The membrane was washed several times with Western Breeze Wash Solution (Invitrogen) at RT. Thereafter, the membrane was incubated with Secondary Antibody Solution (Invitrogen) for 30 min at RT. After additional washes, leptin and β-actin proteins were visualized using Western Breeze Chemiluminescent Substrate (Invitrogen) and ECL mini-camera (Amersham Pharmacia Biotech, Poole, UK).

### Wound healing assay

The effect of leptin on the migration of human epidermal keratinocytes was analyzed with a CytoSelect Wound Healing Assay kit (Cell Biolabs Inc., San Diego, USA). The assay was performed according to the manufacturer’s instructions. In brief, wound healing inserts were put into 24-well cell culture plates. The cells were added to either side of the insert and incubated overnight to form a monolayer. Inserts were removed generating a 0.9-mm open wound field in the monolayer of cells, and then the cells were treated with or without 100 ng/mL of leptin. Images of wound healing were captured using a phase-contrast microscope at 0, 3, 6, 9, 12, 18, and 24 hours after the removal of the inserts. The area of open wound field was calculated by using ImageJ software [26].

### Statistics

All data are expressed as the mean ± standard error (SE). Statistical analysis was performed using the Kruskal-Wallis *H* test and Scheffe’s test. Values of P < 0.05 were considered statistically significant.

## Results

### Expression of Ob-R in human and mouse skin

Immunohistochemical analysis of the expression of Ob-R in human skin showed that Ob-R was expressed in epidermal cells (Fig. 1A and 1B). Another immunohistochemical analysis of mouse skin revealed that leptin was also expressed in prickle and granular cells of epidermis (Fig. 1C and 1D). In mouse skin, the expression of Ob-R was also noted in some epithelial cells of hair follicles (Fig. 1E and 1F).

### Effect of leptin on wound healing in the mouse skin

**Fig 2:**
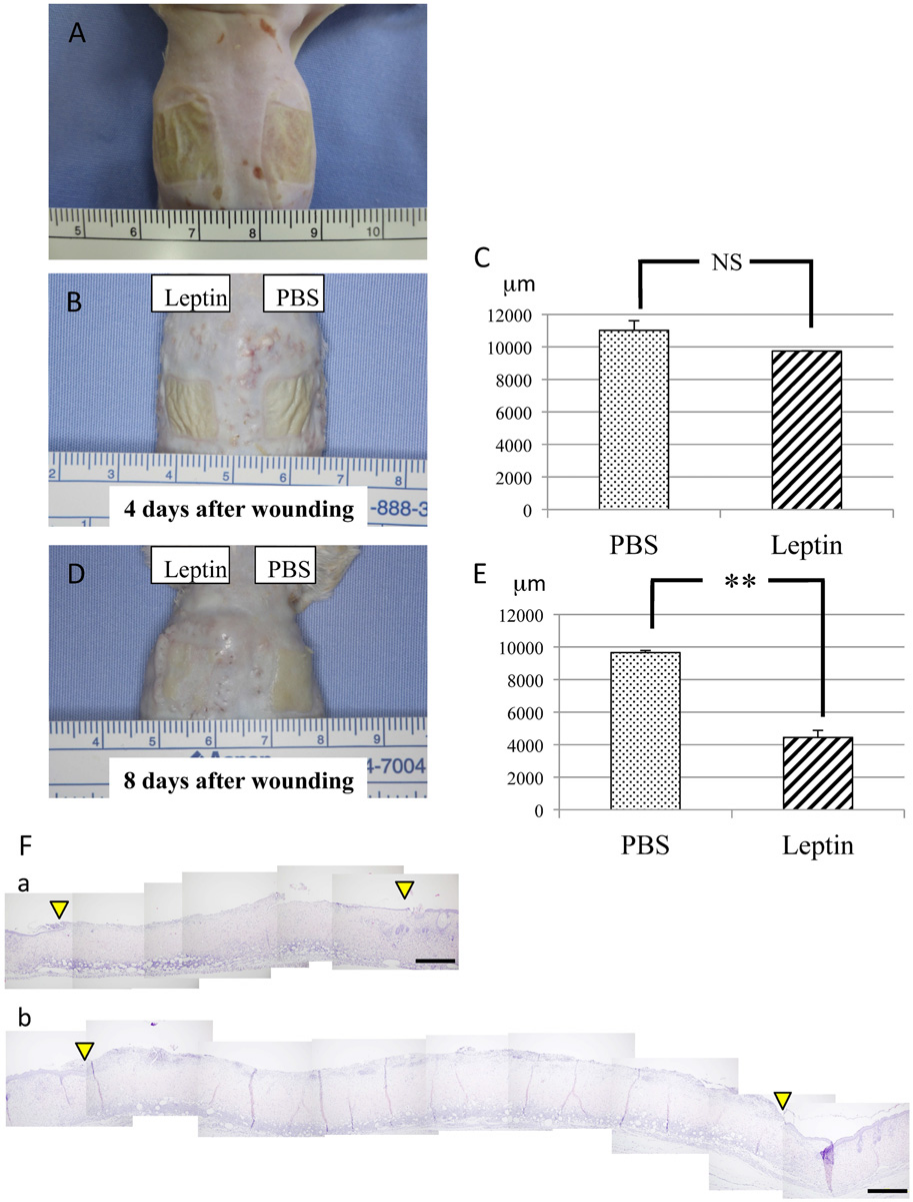
Effect of leptin on wound healing in mouse skin. (A) Chemical wounds created in mouse back skin by applying two pieces of filter paper (12x12mm each) soaked with 20% sodium hypochlorite for 5 minutes. (B and C) Skin wound healing at day 4 after wound creation. No significant difference in wound healing was noted between leptin-treated group and control group. Values are mean ± SE from 12 animals per group. (D and E) Skin wound healing at day 8 after wound creation. Significantly enhanced re-epithelialization of the wound was observed in leptin-treated group compared with control group. Values are mean ± SE from 12 animals per group. **P < 0.01. (F) Histological findings during wound repair at day 8 after wound creation. Spaces between the two arrowheads show ulcerated area without epithelial lining. H-E staining. (a) Leptin-reated group. (b) Control group. Scale bars = 500 μm.

**Fig 3:**
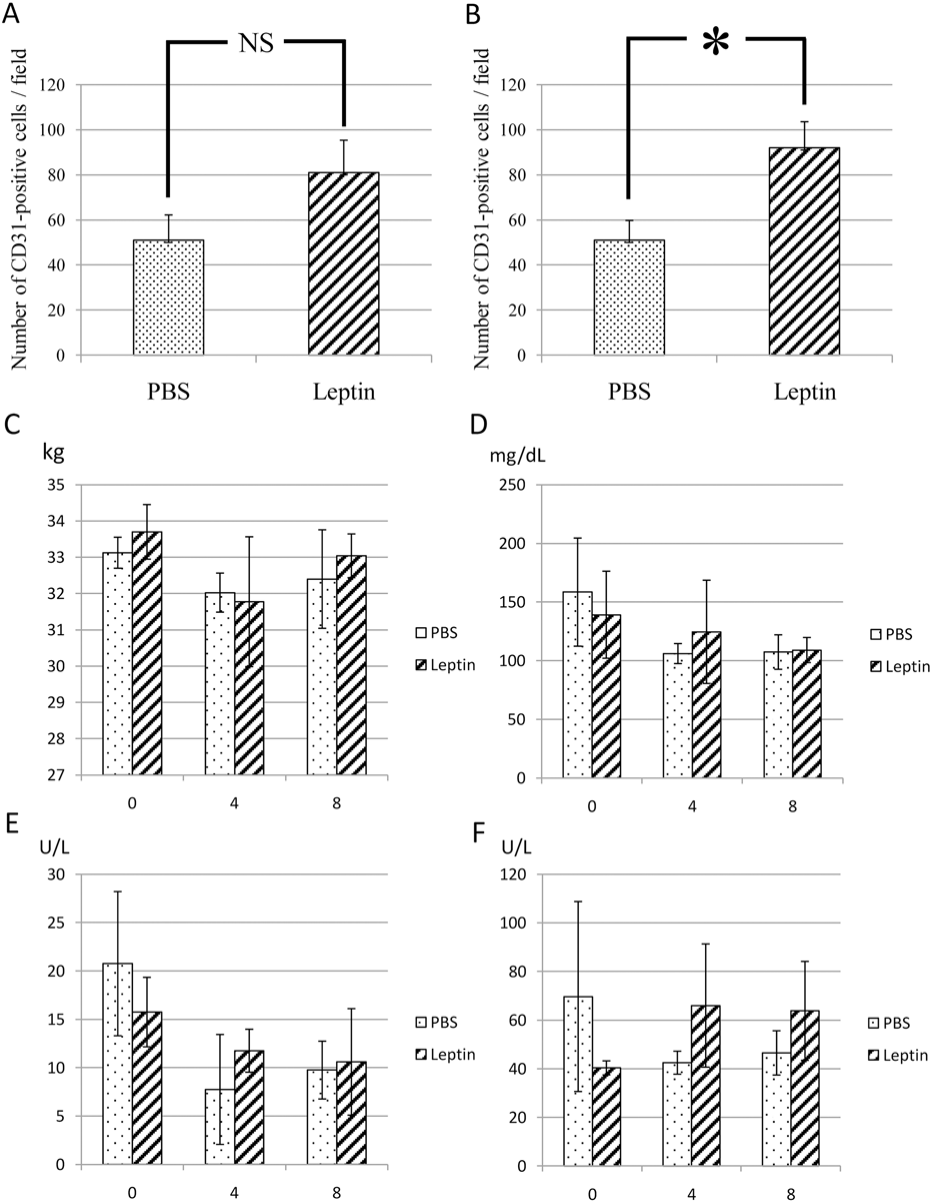
Number of blood vessels in the dermal connective tissue beneath the ulcerated area. (A) At 4 days after initial wounding, no significant difference in the number of CD31-positive cells between Leptin-treated group and control group. (B) At 8 days, more CD31-positive cells were observed in the dermal connective tissue beneath the ulcerated area of leptin-treated group compared with control group. Values are mean ± SE from 5 animals per group. *P < 0.05. (C) Changes in body weight. (D) Changes in serum levels of blood sugar. (E) Changes in serum levels of AST. (F) Changes in serum levels of ALT. None of these laboratory parameters were significantly affected by leptin application. Values are mean ± SE from 5 animals per group.

To elucidate the effect of topically administered leptin on wound healing in the skin, MedGel containing leptin or PBS was directly applied to the skin wounds (Fig. 2A). The size of the ulcer, i.e. the longest span of the ulcer, was measured in addition to histological examination at day 4 and day 8 after wound creation. At day 4 after wound creation, no significant difference in wound healing was noted between two groups (Fig. 2B and 2C). In contrast, at day 8 after wound creation, significantly enhanced re-epithelialization of the wound was observed in leptin-treated group compared with control group (Fig. 2E-F). Histometric analysis of dermal connective tissue revealed that more CD31-positive cells, i.e. more blood vessels, were observed in the connective tissue beneath the ulcer area in the leptin-treated group compared with the control group at day 8 after would creation (Fig. 3A and 3B). Meanwhile, body weight, and levels of AST, ALT or BS in sera were not affected by leptin administration throughout the whole experimental period, which suggests that topically administered leptin caused no systemic adverse effects (Fig. 3C-F).

### Effects of leptin on human epidermal keratinocytes

**Fig 4:**
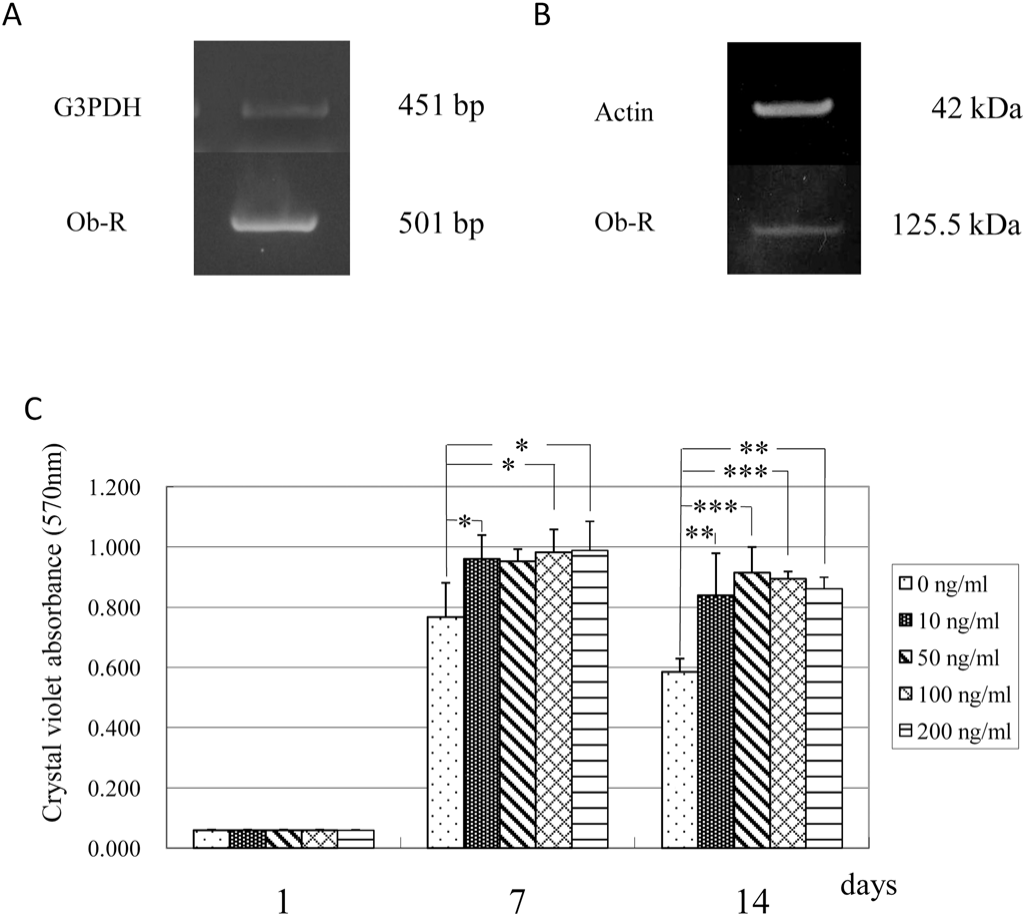
Effect of leptin on human epidermal keratinocytes. (A) *Ob-R* mRNA expression in human epidermal keratinocytes. (B) *Ob-R* protein expression in human epidermal keratinocytes. (C) Effect of leptin on the proliferation of human epidermal keratinocytes. Leptin enhanced cell proliferation at a concentration equal to and more than 10 ng/mL. Values are mean ± SE (n = 8). *P < 0.05. **P < 0.01. ***P < 0.001.

The expression of *Ob-R* mRNA and protein in human epidermal keratinocytes was confirmed using RT-PCR analysis and Western blot analysis, respectively. These experiments showed that human epidermal keratinocytes expressed mRNA and protein for *Ob-R* (Fig. 4A and 4B). To elucidate the effect of leptin on the proliferation of these cells, the cells were cultured in the absence or presence of various concentrations (10, 50, 100 and 200 ng/mL) of leptin. The results indicated that the proliferation of human keratinocytes was significantly enhanced by leptin at a concentration equal to and more than 10 ng/mL (Fig. 4C).

**Fig 5:**
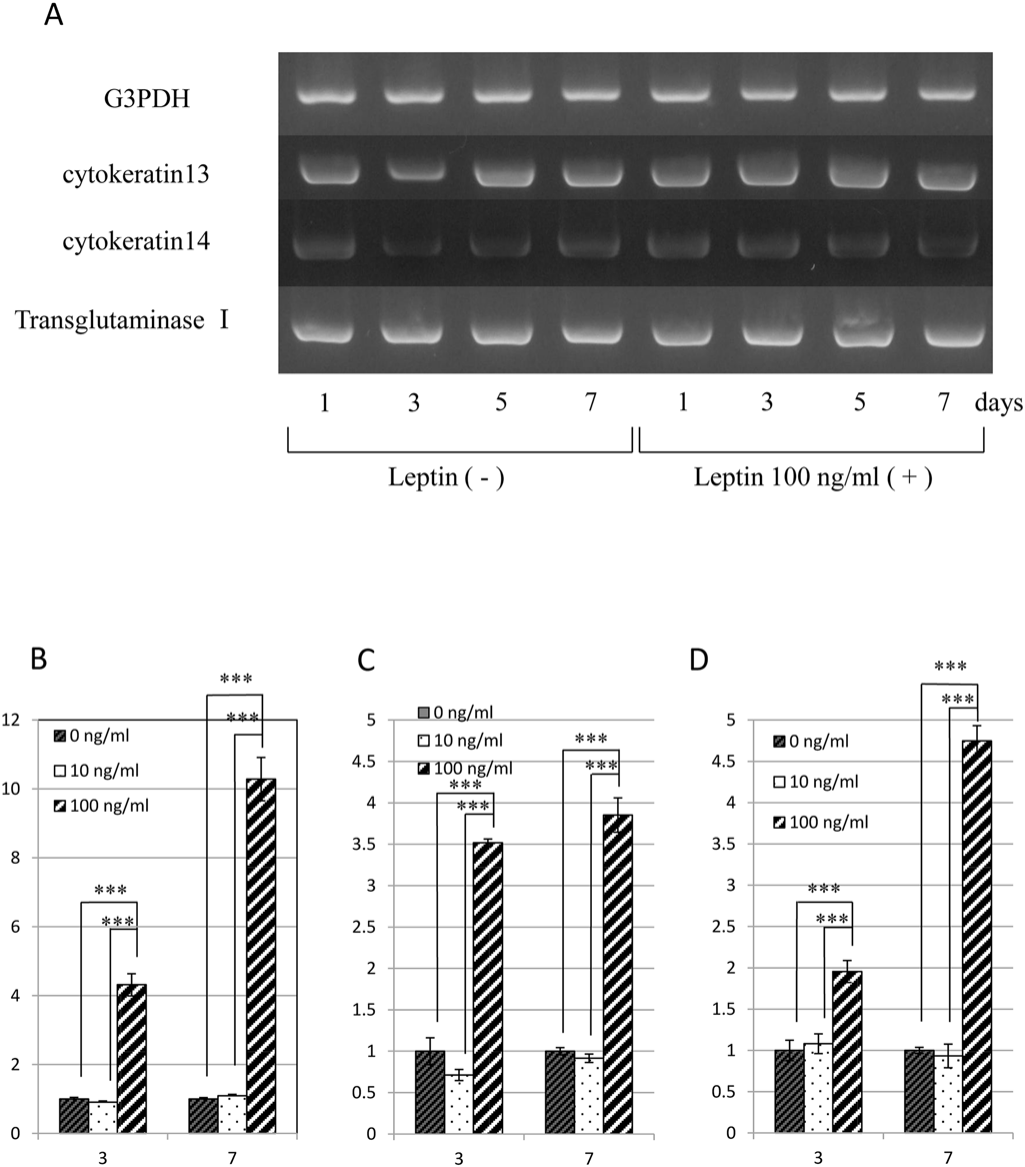
Effect of leptin on the expression of mRNA encoding *G3PDH* and *Cytokeratin 13*, *Cytokeratin 14* and *Transglutaminase I* in human epidermal keratinocytes. (A) Gene expression was analyzed by semi-quantitative RT-PCR analysis. (B-D) Effect of leptin on the expression of mRNA encoding *Cytokeratin 13* (B), *Cytokeratin 14* (C) and *Transglutaminase I* (D). Gene expression was analyzed by quantitative RT-PCR analysis. Values are mean expression ± SD of each gene (n = 8). ***P < 0.001.

Next, the effect of leptin on the differentiation/function of human keratinocytes was investigated using semi-quantitative RT-PCR analysis of the expression of mRNA encoding kereatinocyte-related genes, i.e. *Cytokeratin 13*, *Cytokeratin 14* and *Transglutaminase I*. No apparent effect of leptin on these cells was noted from this analysis (Fig. 5A). In contrast, however, quantitative RT-PCR analysis performed to evaluate putative differences in these gene expressions revealed that leptin exerted stimulatory effect on the gene expression of *Cytokeratin 13*, *Cytokeratin 14* and *Transglutaminase I* at the concentration of 100 ng/mL (Fig. 5B-D).

**Fig 6:**
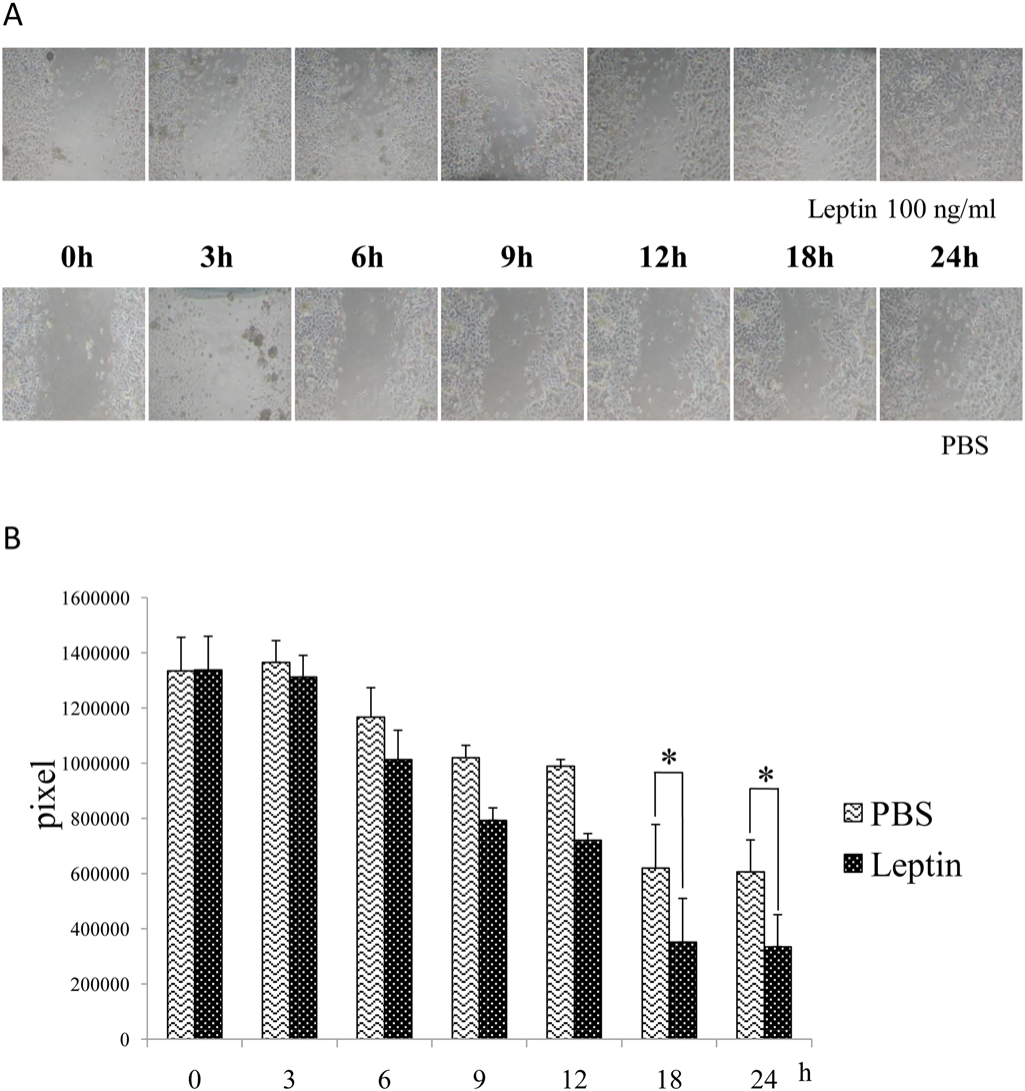
Effect of leptin on the migration of human epidermal keratinocytes. Leptin enhanced the migration of cells. Values are mean ± SE from two independent experiments performed in triplicate. *P < 0.05

Finally, to elucidate the effect of leptin on the migration of human epidermal keratinocytes, a wound healing assay was performed. Interestingly, this assay revealed that leptin significantly accelerated the migration of human epidermal keratinocytes (Fig. 6).

## Discussion

Leptin is widely known as an anti-obesity hormone produced and secreted mainly by adipose tissue [1]. Accumulating evidence shows that leptin is also produced/secreted by a variety of tissues/cells such as placenta [11], skeletal muscles [13] and brain [15] other than adipose tissue and that Ob-R, a most potent specific receptor, also expressed various cells/tissues such as hypothalamus [17,18], hepatocytes [19,20] and endothelial cells [22,23]. These findings, i.e. wide distribution of both production sites and target sites strongly suggest the multifunctionality of leptin. In fact, leptin exerts its influence on a variety of physiological events such as hematopoiesis [4], bone formation [7,8] and angiogenesis [9,10].

Leptin-deficient *ob/ob* mice have been used as a model system to investigate the cellular and molecular mechanisms of impaired wound healing. The severe impairment of wound healing observed in these model mice was originally explained by the diabetic phenotype of the animals. However, Frank *et al*. demonstrated that systemically and topically applied leptin markedly improved re-epithelialization of excisional skin wounds in *ob/ob* mice and that topically applied leptin accelerated normal cutaneous wound healing even in wild-type mice [22]. In this study, they topically applied leptin by covering the skin wound with 1 μg leptin in 20 μL PBS (for *ob/ob* mice) or 5 μg leptin in 20 μL PBS (for wild-type mice) twice a day for 13 days. In addition, Ring *et al*. also demonstrated that systemically and topically administered leptin both accelerate wound healing in diabetic *ob/ob* mice [27]. In this study, they directly applied 1 to 30 μg leptin to the wound every day for 8 days, confirmed that topical administration of leptin promote the wound healing more quickly compared with the systemic administration. In our previous study, we also demonstrated that topically-administered leptin promoted the wound healing in the oral mucosa [23]. The findings obtained from these investigations strongly suggest the definite possibility of leptin as a wound healing-promoting agent. However, in actually using leptin as a wound healing-promoting agent in the clinical practice, it is feared that leptin administered to the whole body or administered topically many times may cause some sort of adverse effect. Therefore, in this study, we investigated whether leptin could exert a promotive effect on the skin would healing even administered in a single dose with a low dosage by using MedGel, a bioabsorbable hydrogel used for drug delivery. The skin was much more suitable for the usage of MedGel compared with the oral mucosa, because it was dry enough that MedGel could be easily and stably applied. This was the reason why we employed the skin wound healing assay rather than oral mucosal wound healing assay in this study.

First, the expression/localization of Ob-R in mouse and human skin was immunohistochemically confirmed. This analysis revealed that Ob-R was expressed in the epidermal cells of human skin and in the prickle/granular cells in epidermis of mouse skin. Interestingly, some epithelial cells of hair follicles were also positive for Ob-R. These findings suggest that epidermal cells and hair follicle cells are target cells of leptin.

Next, we investigated the effect of leptin on wound healing in the mouse skin. These experiments showed that the wound area decreased much faster in the leptin-treated group compared with the control group. BW, and levels of AST, ALT or BS were not significantly affected by topical administration of leptin throughout the experimental period. These findings indicate that topically administered leptin is capable of promoting wound healing of the skin without any systemic adverse effects. Histometric analysis of wounded skin showed that significantly more blood vessels were distributed in the connective tissue beneath the ulcer in the leptin-treated group compared with the control group. This finding strongly suggests that leptin stimulates angiogenesis in the connective tissue beneath the ulcer, and promotes wound healing in the skin by accelerating the supply of nutrients, oxygen and even some bioactive substances. This result was consistent with some previous studies on the wound healing of rabbits [23] and rats [28].

To investigate another possible mechanism underlying the promotive effect of leptin on skin wound healing, cell biological analyses were performed using human epidermal keratinocytes proven to express *Ob-R* by RT-PCR analysis and Western blot analysis. The cell proliferation assay showed modest stimulatory effect of leptin on the proliferation of human keratinocytes at concentration equal to and more than 10 ng/mL. Quantitative RT-PCR analysis was performed to examine whether leptin has any influence on the differentiation/function of keratinocytes. This analysis detected an elevation in expression levels of mRNA encoding *Cytokeratin 13*, *Cytokeratin 14*, and *Transglutaminase I* in the presence of 100 ng/mL leptin, suggesting that leptin has a stimulatory effect on the differentiation/function of human keratinocytes. To examine the effect of leptin on cell migration, a wound healing assay was also performed. This assay revealed that leptin promoted the migration of keratinocytes. From these findings, we conclude that leptin is capable of promoting wound healing in the skin by stimulating both proliferation and migration of human epidermal keratinocytes. Some previous studies reported that leptin induced the proliferation of various other cells such as skin keratinocytes [22,29], lung epithelial cells [30], hemopoietic cells [4], pancreatic beta cells [31,32] and endothelial cells [9]. Consistent with these reports, the present study showed a stimulatory effect of leptin on the proliferation of human epidermal keratinocytes. Interestingly, in contrast with this, our previous study demonstrated that leptin affected no significant effect on proliferation or differentiation/function of human oral mucosal epithelial cells [23]. Although there are no clues to explain the reason/mechanism of this difference, this discrepancy may come from the distinctive characteristics of oral mucosal epithelial cells. This issue should be solved in the future study.

The available evidence suggests several possibilities regarding the mechanisms by which leptin promotes wound healing. One possibility is that leptin promotes wound healing by enhancing the epithelial cell proliferation [24,29]. Another possibility is that leptin promotes it by stimulating the angiogenesis [9,10,28]. Interestingly, another previous study showed that leptin was induced in wound tissue during the first few days following injury and affected wound repair process [33]. This fact supports the possibility that leptin palys a physiological role in wound healing. In addition, the accumulating evidence suggested that the skin may be an important peripheral neuro-endocrine-immune organ that is tightly networked to central regulatory system and contribute to the maintenance of peripheral homeostasis [34–38]. In this context, wound healing in the skin is considered to be a very dynamic and complex process involving a variety of hormones and cytokines. In this study, unfortunately, we could not elucidate the overall view of wound healing in the skin. This issue should be elucidated in the future investigation. Moreover, some findings in the present study were obtained from the animal experiments. So, it is definitely important to confirm the physiological/pharmacological effect of exogenously-administered leptin in humans. However, judging from the behavior of human epidermal keratinocytes, it would be very promising that topically-administered leptin could promote wound healing in the human skin. This issue could be resolved in the future clinical trials.

In the present study, nevertheless, we clearly demonstrated for the first time that leptin promotes wound healing in the skin by accelerating the migration of epidermal cells. Moreover, we showed that topically administered leptin could promote wound healing in the skin without any side effects. Judging from the multifunctionality of leptin, a systemic administration or multiple local administration of leptin may cause some kind of adverse effects in its clinical application. From this point of view, a single dose topical administration is considered to be more advantageous than systemic or multiple local administration. So we here investigated whether leptin exerted a promotive influence on the skin would healing even when administered with a low single dosage and one time by using MedGel.

Taken together, we concludes that leptin is capable of accelerating wound healing in the skin by promoting angiogenesis around the wounded area and by enhancing the proliferation, differentiation/function and migration of epidermal keratinocytes. Importantly, our study also clearly demonstrated that leptin is effective for wound healing acceleration even in a single dose when applied topically by using an adequate drug delivery system. It is still necessary and important, however, to judge carefully whether local administration or systemic administration of leptin is more advantageous in the clinical practice. Further studies would be required to resolve this issue. Nonetheless, the present study is considered to pave the way for the clinical utilization of leptin as a wound healing-promoting agent.
